# N-WASP deficiency enhances oncogenic KRAS-mediated lung adenocarcinoma progression in mice

**DOI:** 10.3389/fonc.2026.1868586

**Published:** 2026-10-08

**Authors:** Apoorva Verma, Nanyang Li, Pazhanichamy Kalailingam, Chaiyoung Kim, Ankur Sharma, Ying Hui Lee, Thirumaran Thanabalu

**Affiliations:** School of Biological Sciences, Nanyang Technological University, Singapore, Singapore

**Keywords:** actin cytoskeleton, Erk2 signaling, KRAS lung cancer, N-WASP, transgenic mice, tumor suppressor

## Abstract

**Objective:**

Oncogenic KRAS mutations drive 25-30% of western lung adenocarcinoma (LUAD) cases, however the mechanisms that restrain tumor progression remain poorly defined. N-Wiskott–Aldrich syndrome protein (N-WASP) regulates actin cytoskeleton dynamics and epithelial architecture, but its role in KRAS-driven lung tumorigenesis is unclear.

**Methods:**

We generated a conditional N-WASP knockout in KRAS^G12D^-driven lung adenocarcinoma mice (N-WASP^LKO^/KRAS^G12D^) using Sftpc-CreERT2, and assessed N-WASP knockdown A549 cells (A549^KD^) in 3D culture for tumor burden, ERK signaling, transcriptomic changes, and E-cadherin expression.

**Results:**

Conditional deletion of N-WASP in type II alveolar cells (N-WASP^LKO^) markedly increased tumor number and size in KRAS^G12D^ mice. The tumors isolated from these mice retained strong pro–surfactant protein C expression, confirming alveolar epithelial origin and predominantly adopted a solid growth pattern, indicative of disrupted epithelial organization. Mechanistically, N-WASP loss enhanced KRAS-driven ERK1/2 activation in lung tissue, consistent with increased mitogenic signaling. In A549 cells, N-WASP deficiency disrupted cytoskeletal architecture, promoted proliferation, and impaired cell–cell adhesion, recapitulating *in vivo* tumor phenotypes. A kinase inhibitor screen identified Rho-associated protein kinase (ROCK) activity as a driver of this hyperproliferative phenotype, with ROCK inhibition normalizing proliferation in N-WASP-deficient cells, linking elevated Rho/ROCK signaling to the increased ERK1/2 activation observed upon N-WASP loss. Transcriptomic profiling of N-WASP–deficient lungs revealed coordinated upregulation of genes regulating proliferation, cytoskeletal remodeling, and tumor-promoting pathways, alongside downregulation of epithelial differentiation and adhesion programs.

**Conclusion:**

These results establish N-WASP as a critical suppressor of KRAS-driven lung adenocarcinoma, linking cytoskeletal integrity to oncogenic signaling and transcriptional regulation. Our findings highlight the interplay between actin dynamics, epithelial architecture, and KRAS signaling, suggesting that targeting N-WASP dependent pathways could modulate lung cancer aggressiveness and offer novel therapeutic opportunities.

## 1 Introduction

Lung cancer is the leading cause of death worldwide with an estimated 2 million deaths each year and represents 18% of all cancer associated deaths (1). Lung cancer is classified into Non-Small Cell Lung Cancer (NSCLC) and Small Cell Lung Cancer (SCLC). NSCLC accounts for 85% while SCLC accounts for the remaining 15% of all lung cancer (2). Oncogenic mutations in KRAS represent one of the most frequent genetic alterations in human cancers, particularly in lung, colorectal and pancreatic malignancies (3). In lung adenocarcinoma (LADC), KRAS mutations occur in approximately 30% of cases and are strongly associated with aggressive disease and limited therapeutic options (4).

Actin cytoskeleton remodeling plays a critical role in epithelial cell proliferation, migration, elongation, and branching morphogenesis which are essential for proper lung development (5–7). The actin cytoskeleton is made of G-actin (Globular) and F-Actin (Filamentous) and the rate limiting step in polymerization of G-actin to F-actin is nucleation. Small Rho GTPases, particularly Cdc42, play a central role in orchestrating actin dynamics by activating downstream effectors. Genetic ablation of Cdc42 results in profound defects in branching, polarity, and spatial organization, ultimately leading to perinatal lethality, underscoring the importance of actin regulatory pathways in lung development (8). One of the downstream effectors of Cdc42 is the ubiquitously expressed N-WASP which activates the Arp2/3 complex (actin filament nucleation factor) thus plays a vital role in actin cytoskeleton remodeling (9). Upon activation by active Cdc42, N-WASP activates Arp2/3 complex, thereby regulating epithelial architecture and tissue morphogenesis. N-WASP has been shown to be essential for the formation of actin rich structures such as invadopodia and filopodia in metastatic carcinoma cells (10) and invadopodium formation mediated by N-WASP is crucial in breast cancer invasion, intravasation, and lung metastasis (10, 11). Constitutive knockout of N-WASP in mice caused defects in neural tube formation, cardiac tissue and embryonic lethality (12). N-WASP regulates cell-ECM adhesion as mouse embryonic fibroblast lacking N-WASP adhered poorly to fibronectin coated surface (13).

Dysregulation of actin remodeling pathways is increasingly recognized as a key driver of cancer progression, invasion, and metastasis. Notably, reduced expression of N-WASP has been reported in several cancers, including breast, colorectal, and lung cancers, where it is associated with poor patient prognosis (14–16). In contrast, emerging evidence suggests a more complex, context-dependent role for N-WASP in tumorigenesis. For example, loss of N-WASP accelerates intestinal tumor formation in an APC-mutation driven colorectal cancer (17), whereas increased N-WASP expression has been observed in late-stage lung cancer and correlates with reduced patient survival (18). These findings suggest that N-WASP may function as a stage- and tissue-specific modulator of cancer progression. While the role of KRAS signaling pathways in cell proliferation and survival is well characterized, its influence on actin cytoskeleton remodeling and epithelial architecture through downstream effectors such as N-WASP remains incompletely understood.

In this study, we investigate the functional interplay between oncogenic KRAS signaling and N-WASP mediated actin regulation in lung adenocarcinoma. By generating a conditional N-WASP knockout in a KRAS^G12D^ -driven lung adenocarcinoma mouse model, we assess the impact of N-WASP loss on tumor initiation, growth, and progression *in vivo*. Complementary *in vitro* analyses using A549, a human alveolar adenocarcinoma cell line with a gain-of-function mutation KRAS^G12S^ further delineate the cellular and molecular mechanisms underlying N-WASP- dependent tumor behavior. Together, this work positions N-WASP as a critical downstream effector in KRAS-driven lung tumorigenesis and highlights cytoskeletal remodeling pathways as potential therapeutic targets in KRAS-mutant lung cancer.

## 2 Materials and methods

### 2.1 Animals and husbandry

Sftpc-CreER^T2^ knock in mice (Stock no: 028054), KRAS^LSL-G12D^ mice (Stock no: 008179), R26-stop-EYFP mutant mice (Stock number: 006148) from Jackson laboratory, USA were used together with our laboratory generated N-WASP^fl/fl^ mice (exons 3 & 4 of N-WASP gene flanked with loxp-loxp sites) (19) to generate the mice used in this study. The mice were maintained in the NTU animal facility on a balanced diet with unlimited water. All mice experiments were performed according to approved protocols of Institutional Animal Care and Use Committee (A0386 and A18020).

### 2.2 Mouse tail PCR genotyping and tamoxifen injection

4 weeks old mice were given tamoxifen through intraperitoneal injection at a dose of 75mg/kg body weight for 5 consecutive days. The mice generated were genotyped as described previously (20). Briefly, genomic DNA was extracted from 2-5mm tail tip biopsies by overnight digestion in tail lysis buffer (20). PCR amplification was performed using the KAPA HiFi PCR Kit (Kapa Biosystems, Wilmington, DE, USA) as per manufacturer’s instructions. The list of primers used for mouse tail PCR genotyping is shown in **Table 1**.

**Table 1:** I Primer sequences used for mouse tail PCR genotyping.

Primer name	Forward primer 5’ to 3’ (common)	Reverse primer 5’ to 3’ (wild type)	Mutant
LoxP	AGCTCAGAGAAGGTGTATTGG	GCTCTTGTTCCCACAGTAATTTCC	
Sftpc Cre	TGCTTCACAGGGTCGGTAG	CATTACGTGGGGTAGGACCA	ACACCGGCCTTATTCCAAG
KRas	AGCTAGCCACCATGGCTTGAGTAAGT	GTCTTTCCCCAGCACAGTGC	CTCTTGCCTACGCCACCAGCT

### 2.3 Histological analysis

Tissues isolated from the mice were fixed with 4% paraformaldehyde at 4 °C overnight, washed with 1x PBS, processed and embedded as described previously (20). The embedded tissues were sectioned into 5μm size and transferred onto Superfrost Plus slides (Fisher Scientific, USA) and stained with haematoxylin and eosin (H&E) (20).

### 2.4 Protein extraction from lung tissue and western blot analysis

Whole-lung protein lysates were prepared by finely mincing lung lobes with sterile scissors, followed by mechanical homogenization using a syringe plunger in protein lysis buffer (21). The homogenates were maintained on ice for 1 hour and subsequently centrifuged at 15,000 x *g* for 15 mins at 4 °C. The protein concentration of the supernatant was quantified using Bradford assay and resolved on SDS-PAGE for western blot analysis. The antibody dilution used for western blot are as follows: anti-N-WASP (laboratory generated, 1:1,000), anti-GAPDH (Ambion, 1:1, 000), anti-KRas^G12D^ (Cell Signalling, 1:1,000), anti-ERK1/2 (Cell Signalling, 1:500), anti-phospho Thr202/Tyr204 ERK1/2 (Cell Signalling, 1:1,000), anti-cyclin D1 (Santa Cruz, 1:1,000), secondary antibodies conjugated with horseradish peroxidase, anti-mouse and anti-rabbit (Sigma, 1:10,000).

### 2.5 Isolation of lung epithelial cells and sorting of type II alveolar cells

Lung epithelial cells were isolated as described in (22) with few modifications. Mice were sacrificed using CO_2_ inhalation. After the lungs and heart of the mice were exposed, perfusion was carried out by injecting 1x PBS in right bottom ventricle by using 30G needle with 10ml syringe. The lungs were inflated with 2ml dispase solution (5 mg/ml) through the trachea, isolated and kept in sterile Eppendorf tube. The lungs were chopped using scissors in 1ml warm media (DMEM + 2% FBS) and incubated with collagenase and DNAse. DMEM + 2% FBS was added to top the volume to 2 ml and incubated at 37°C for 60 mins with gentle agitation. The lung tissue lysates were homogenized by passing through 18.5G needle. The cell lysate was passed through cell strainer (40 μm) and the cell suspension was collected and centrifuged at 350 x *g* for 5 mins. The pellet was incubated with red blood cells lysis buffer and mixed by gentle inversion. The tubes were incubated for 10 mins at room temperature after which they were spun down. The pellet was resuspended in 3–5 ml DMEM + 2% FBS media.

The Type II alveolar cells were sorted using BD FACS Aria Cell Sorter (BD Biosciences, Franklin Lakes, NJ, USA). FACS antibody cocktail mix was prepared using PE anti-Epcam (Biolegend, 1:1,000) and APC/FIRE anti-CD45 (Biolegend, 1:600) in 1x PBS containing 2% FBS. The cells were incubated in antibody solution for 30 mins at 4°C. The cells were collected by centrifugation, and the cells were resuspended in 1x PBS containing 2% FBS in FACS tubes and sorted at the FACS facility. The CD45 negative population was collected and sorted for Epcam positive. The sorted cells were collected in FACS tube after which they were checked for purity by staining with ProSP-C (Millipore, 1:100). The cells were used to isolate RNA for RNA sequencing.

### 2.6 RNA sequencing and analysis

Total RNA from sorted Type II alveolar cells was isolated by Trizol method. The RNA concentration was determined and 2ug of RNA was shipped in RNA Stable tubes on dry ice. The RNA integrity number (RIN) was analyzed using Agilent 2100. The RNA sequencing was performed by NovogeneAit (A*Star, Singapore). The mRNA was enriched using oligo (dT) beads and fragmented randomly into short fragments (~200bp) and 1^st^ cDNA strand was synthesized by using random hexamer primers, after which a custom second-strand cDNA was synthesized using buffer (Illumina), dNTPs, RNaseH and DNA polymerase I. The libraries were fed into Illumina HiSeq platform. DEGs were identified by aligning RNAseq against Mus musculus reference genome. RNA sequencing was performed on two independent biological replicates per genotype (n = 2 per group); given this sample size, the transcriptomic findings reported below should be regarded as exploratory and hypothesis-generating rather than confirmatory. The RNAseq data files are available at GSE343586.

### 2.7 Immunofluorescence staining

The paraffin slides were incubated at 60°C (2 hours) to dissolve the paraffin and were subsequently rehydrated by stepwise incubation from xylene to ethanol to water. Antigen retrieval was performed by heating the slides at 95°C (10 mins) in 0.1M sodium citrate buffer (pH 6.0). The slides were cooled down, rinsed with 1x PBS and incubated for 1 hour in PBS/3% BSA at room temperature. Primary Ab (1:50 or 1:100) diluted in 3% BSA was added to slides, covered with parafilm, placed in a moist chamber and incubated overnight at 4°C. The slides were then washed 3 times with 0.1% PBST and incubated with secondary antibody (Alexa flour 488 or 594, Molecular Probes) and DAPI (Molecular Probes) diluted (1:500) in 3% BSA. Slides were incubated for 1 hour in the dark at room temperature and subsequently washed 5 times with 0.1% PBST. DAPI (Molecular Probes, 1:500) in 1x PBS and mounted using Dako aqueous mounting media. Fluorescence microscopy was performed using Olympus IX51 microscope and images were acquired using Cool SNAPHQ camera and the MetaVue program (Molecular Devices, USA).

### 2.8 Cell culture and transfection

A549 cell line (Human lung adenocarcinoma) and HEK293T (Human Embryonic Kidney cells) (ATCC, USA) were cultured using DMEM + 10% FBS and 100 IU/ml penicillin and 100 IU/ml streptomycin) at 37°C in a 5% CO_2_ humidified incubator.

N-WASP shRNA (GCACAACTTAAAGACAGAGAActcgagTTCTCTGTCTTTAAGTTGTGC) was cloned into pLJM1, lentiviral plasmid (a gift from David Sabatini; Addgene plasmid # 19319) (23) under the transcriptional regulation of U6 promoter and used to knock down N-WASP expression. Lentivirus particles were generated by transfecting HEK293T cells with lentiviral control plasmid or plasmid expressing human N-WASP specific shRNA together with 3^rd^ generation packaging plasmids. The culture supernatant was collected 24 hours and 48 hours after the transfection, filtered and used to infect A549 cells and selected with puromycin.

### 2.9 Cell lysis and western blot analysis

A549 cells were harvested and lysed using RIPA lysis buffer (24). The protein lysate (30 μg) was resolved using 10% SDS-PAGE for western blotting as described above. Densitometric analysis was performed using Image J software on data obtained from three independent set of experiments.

### 2.10 Spheroid proliferation assay

A549^ctrl^ and A549^KD^ were resuspended at 3 X 10^5^ cells/ml in complete media and 100 μl of cell suspension (containing 30, 000 cells) was dispensed onto the wells of 96 well Ultra Low Attachment plates (ULAP). The plates were incubated for 72 hours, after which the spheroids formed were imaged and incubated with 10 μl of WST-8 (MedChemExpress) for 2 hours. The absorbance was measured at 450nm and 630nm (reference wavelength). The WST-8 signal was corrected for background by subtracting OD_630nm_ reading from OD_450nm_ (Tecan Group Ltd., Switzerland).

### 2.11 Statistical analysis

All experiments were conducted in triplicates and data are presented as mean ± standard deviation of the mean, as indicated in the bar graphs. Statistical significance between control and experimental group was determined using Student’s *t*-test. Significance levels are denoted as follows: * (p<0.05), ** (p<0.01) and *** (p<0.001).

## 3 Results

### 3.1 Conditional ablation of N-WASP in KRAS^G12D^-driven lung cancer increases tumor initiation *in vivo*

To assess the *in vivo* relevance of N-WASP loss in lung tumorigenesis, a conditional N-WASP knockout KRAS^G12D^ mouse model was generated using Sftpc-CreER^T2^ to target expression of oncogenic KRAS^G12D^ and N-WASP ablation in type II alveolar epithelial cells. We generated N-WASP^Het^ (N-WASP^fl/WT^; Sftpc-CreER^T2^) and N-WASP^LKO^ (WASP^fl/fl^; Sftpc-CreER^T2^) mice using established mice breeding techniques. The genotype of the mice generated was verified by PCR using tail genomic DNA (**Supplementary Figure 1**) and primers indicated in methods. Tamoxifen administration in these mice induced simultaneous deletion of N-WASP and activation of oncogenic KRAS^G12D^ expression specifically within this epithelial population (**Supplementary Figure 1**). This approach allowed temporal control of tumor initiation while restricting genetic alterations to a physiologically relevant cell of origin for lung adenocarcinoma.

Western blot analysis of whole lung lysates demonstrated a significant reduction in N-WASP protein expression in knockout mice while validating expression of KRAS^G12D^ (**Figure 1A**). We found that N-WASP^LKO^/KRAS^G12D^ mice developed a significantly higher number of lung tumors compared to heterozygous (**Figure 1B**). Quantitative analysis across cohorts (n = 5 per group) demonstrated a robust increase in tumor multiplicity in N-WASP^LKO^/KRAS^G12D^ mice compared to N-WASP^Het^/KRAS^G12D^ mice (**Figure 1B**). These data establish N-WASP as a negative regulator of KRAS-driven tumor initiation *in vivo*. N-WASP^Het^/KRAS^G12D^ mice were used as the principal comparator for the protein and transcriptomic analyses because, in our hands and in our prior work in other epithelial tissues (20), they are phenotypically indistinguishable from N-WASP^WT^/KRAS^G12D^ mice at this timepoint and provide a litter-matched control that reduces animal numbers consistent with the 3Rs principle of our IACUC protocol; a fully N-WASP-wild-type KRAS^G12D^ control group was additionally included for the tumor number and size endpoints (**Figures 1B**, **2B**) and confirmed that heterozygous loss of N-WASP alone does not significantly alter tumor burden relative to wild-type.

**Figure 1:**
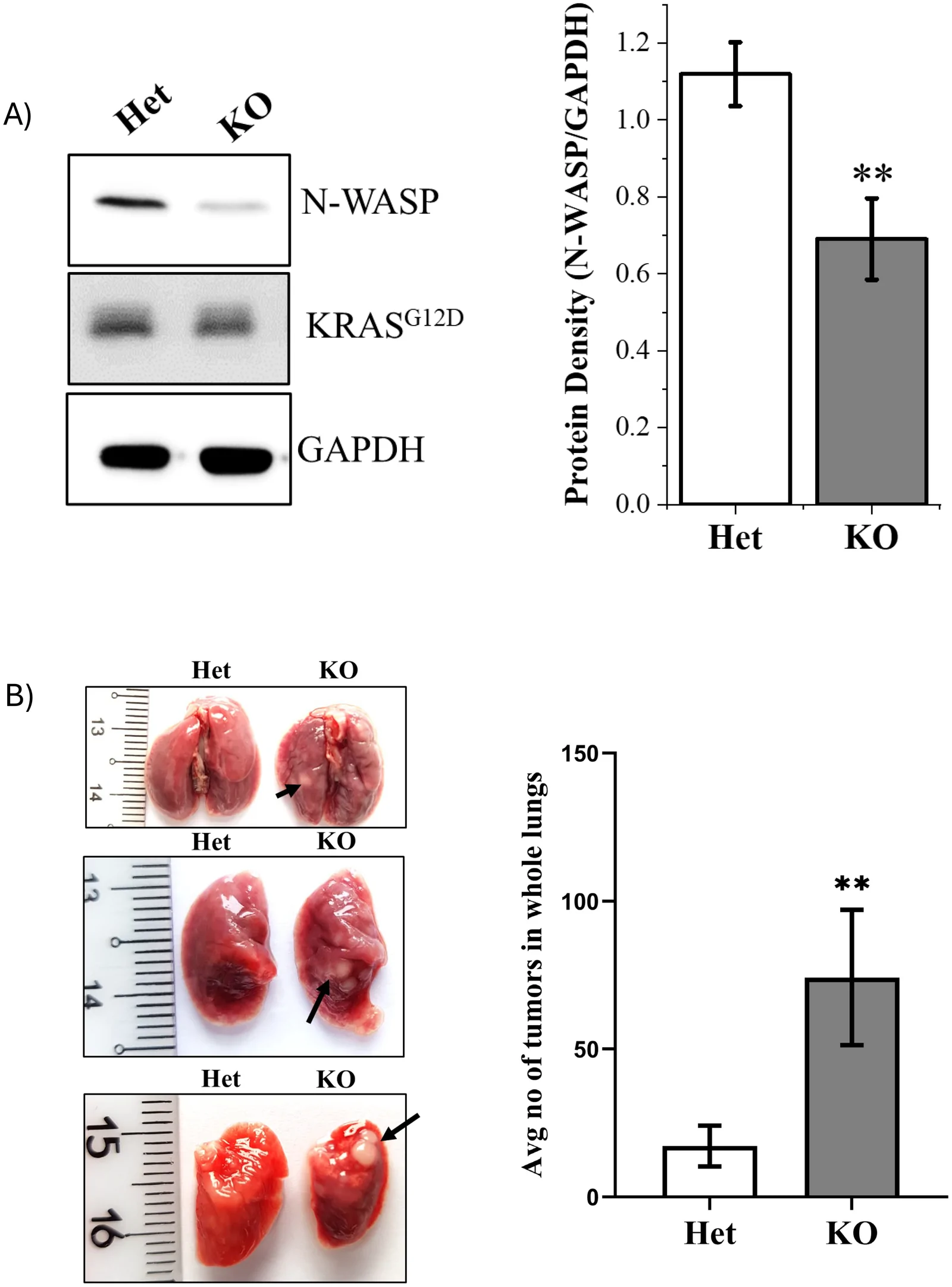
Homozygous N-WASP knockout caused a significant increase in lung tumors in mice expressing oncogenic KRas^G12D^ compared to heterozygous knockout of N-WASP. **(A)** Lungs were harvested from N-WASP^LKO^/KRas^G12D^ (KO) and N-WASP^LHet^/KRas^G12D^ (Het ctrl) mice 5 weeks after tamoxifen injection. The lungs were homogenized, lysed and equal amount of protein (30ug) was run on SDS gel to check for N-WASP and KRas^G12D^ protein level. The graph shows significant reduction in N-WASP in KO compared to control. (n=3 per group) (**p ≤ 0.01) **(B)** The mice were injected with tamoxifen and sacrificed 5 weeks after tamoxifen injection. The lungs were harvested for tumor growth analysis. N-WASP^LKO^/KRas^G12D^ (KO) mice shows significantly higher number of tumors in whole lungs compared to N-WASP^LHet^/KRas^G12D^ (Het ctrl) and N-WASP^LWT^/KRas^G12D^ (WT ctrl) for n=5 per group (**p ≤ 0.01).

**Figure 2:**
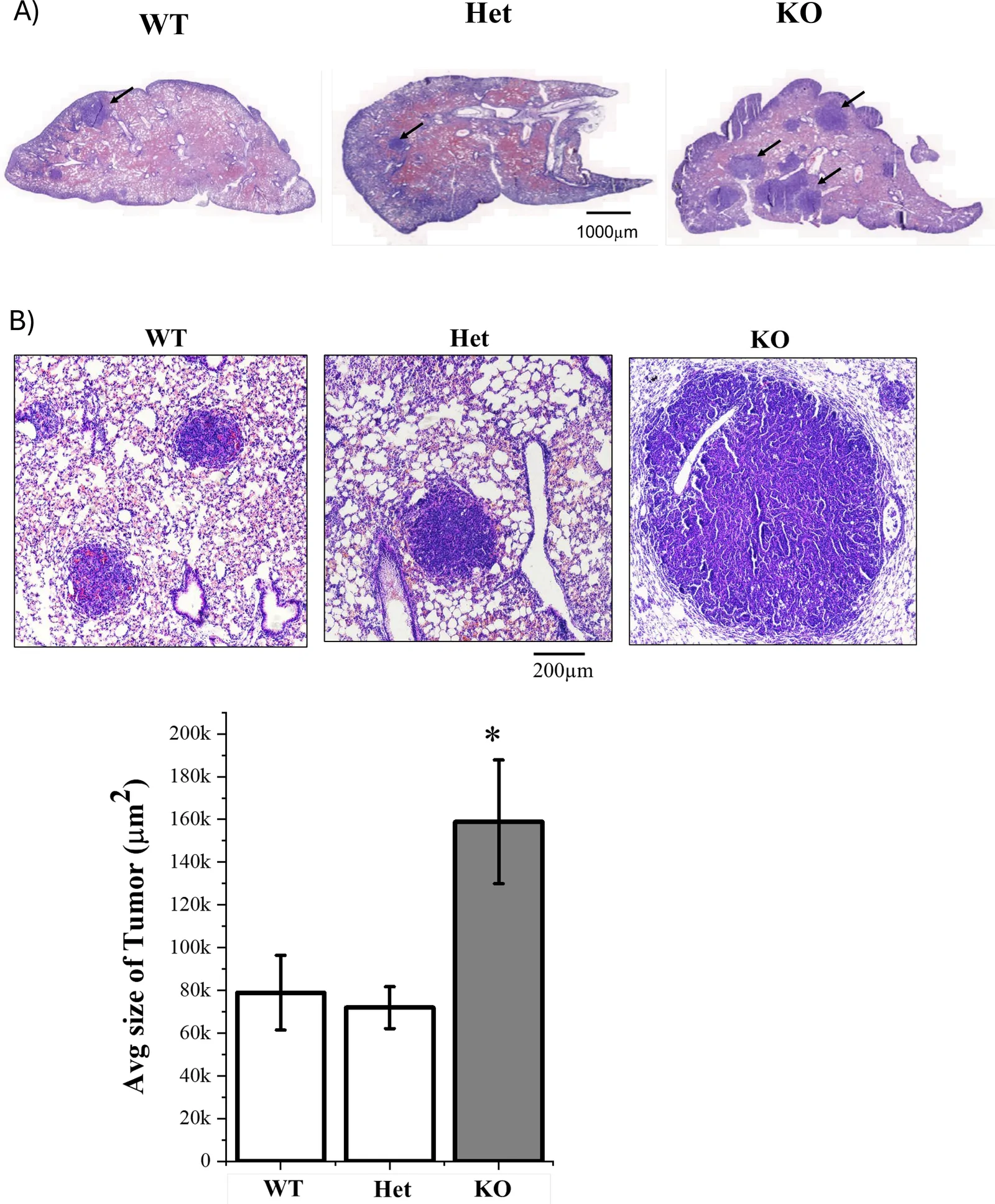
Lungs from N-WASP^LKO^/KRas^G12D^ mice had significantly higher number of tumors and the tumors were significantly larger compared to control. **(A)** All three groups of mice: N-WASP^LKO^/KRas^G12D^ (KO), N-WASP^LHet^/KRas^G12D^ (Het ctrl) and N-WASP^LWT^/KRas^G12D^ (WT ctrl) were injected with tamoxifen and the mice were sacrificed 5 weeks after tamoxifen injection. The left lobes of the lungs were harvested for histopathological analysis using H&E staining. The H&E slides of the left lung lobe were scanned using Axio Scan Z1 slide scanner. N-WASP^LKO^/KRas^G12D^ (KO) mice shows higher number of tumors compared to control (marked by arrows), used n=5 animals per group. **(B)** The left lobes were harvested for histopathological analysis using H&E staining. Images were taken using 10X objective and the area of tumor was quantified using ImageJ at 10X. N-WASP^LKO^/KRas^G12D^ (KO) mice shows larger size of tumors compared to control. (n=5 per group) (Significance is indicated by stars *p ≤ 0.05).

### 3.2 N-WASP loss promotes expansion of type II alveolar cell–derived adenocarcinoma in KRAS^G12D^ lungs

To define the cellular origin and identity of tumors arising upon N-WASP loss in a KRAS^G12D^ background, lung sections were immunostained for pro–surfactant protein C (proSP-C), a well-established marker of type II alveolar epithelial cells (25) and recognized progenitors of lung adenocarcinoma (26, 27). Tumor regions in N-WASP^LKO^/KRAS^G12D^ lungs exhibited strong, widespread proSP-C positivity, confirming their derivation from type II alveolar epithelial cells (**Supplementary Figure 2**). In contrast, non-tumor regions displayed sparse and evenly distributed proSP-C–positive cells, consistent with normal alveolar epithelial organization. These findings demonstrate that loss of N-WASP does not alter tumor lineage commitment but instead enhances malignant expansion within the type II alveolar cell compartment. Histopathological evaluation using H&E staining further revealed marked differences in tumor architecture between genotypes. Tumors arising in N-WASP-deficient lungs predominantly displayed a solid adenocarcinoma growth pattern, characterized by densely packed tumor cells with minimal glandular differentiation and absence of lepidic features (28) (**Figure 2A**). This aggressive histological phenotype contrasts with more differentiated growth patterns observed in control KRas^G12D^ tumors and is indicative of enhanced tumor progression. In addition to altering tumor architecture, N-WASP deletion significantly promoted tumor growth and progression. Quantitative morphometric analysis of H&E-stained lung sections demonstrated that tumors in N-WASP^LKO^/KRAS^G12D^ mice were significantly larger than those observed in KRas^G12D^ control mice (**Figure 2B**). Together with the increased tumor burden, these data indicate that N-WASP deficiency enhances both tumor initiation and subsequent tumor expansion *in vivo*.

Collectively, the combined increase in tumor number, size, and aggressive histological features supports a tumor-suppressive role for N-WASP in KRAS-driven lung adenocarcinoma. By constraining excessive proliferation and preserving epithelial architecture through actin cytoskeletal regulation, N-WASP appears to limit malignant progression downstream of oncogenic KRAS signaling.

### 3.3 Enhanced ERK activation accompanies increased tumor burden in N-WASP-deficient lungs

The pronounced increase in tumor number and size observed in N-WASP–deficient KRAS^G12D^ lungs, suggested augmentation of signaling pathways which regulate cell proliferation, thus we next investigated whether loss of N-WASP augments downstream KRAS signaling *in vivo*. Lung lysates from N-WASP^Het^/KRAS^G12D^ and N-WASP^LKO^/KRAS^G12D^ were analyzed by western blot which demonstrated a marked increase in phosphorylated ERK1 and ERK2 levels in N-WASP^LKO^/KRAS^G12D^ mice compared with heterozygous controls, while total ERK levels remained unchanged (**Figure 3A**). Quantitative densitometric analysis confirmed a significant elevation in ERK activation in knockout lungs (**Figure 3B**), indicating enhanced MAPK pathway signaling. This heightened ERK phosphorylation is consistent with increased mitogenic and pro-tumorigenic signaling and provides a molecular correlation for the accelerated tumor growth and progression observed in the absence of N-WASP. Despite robust activation of ERK, levels of the downstream cell cycle regulator cyclin D1 were not significantly increased in whole lung lysates. This apparent disconnect may reflect several non-mutually exclusive factors, including tumor heterogeneity within lung tissue, dilution of tumor-specific signaling by surrounding non-malignant parenchyma, or altered spatial regulation of ERK signaling outputs. Notably, N-WASP is a key regulator of actin organization and intracellular architecture, raising the possibility that loss of N-WASP disrupts the proper subcellular localization or nuclear transmission of ERK-dependent transcriptional programs rather than uniformly amplifying all downstream targets (29). Together, these data suggest that N-WASP loss enhances KRAS-driven ERK activation *in vivo*, contributing to increased tumor burden, while simultaneously altering the qualitative nature of downstream signaling outputs. This supports a model in which N-WASP functions not only as a structural regulator of epithelial organization but also as a critical modulator of KRAS–ERK signaling amplitude and spatial fidelity during lung adenocarcinoma progression.

**Figure 3:**
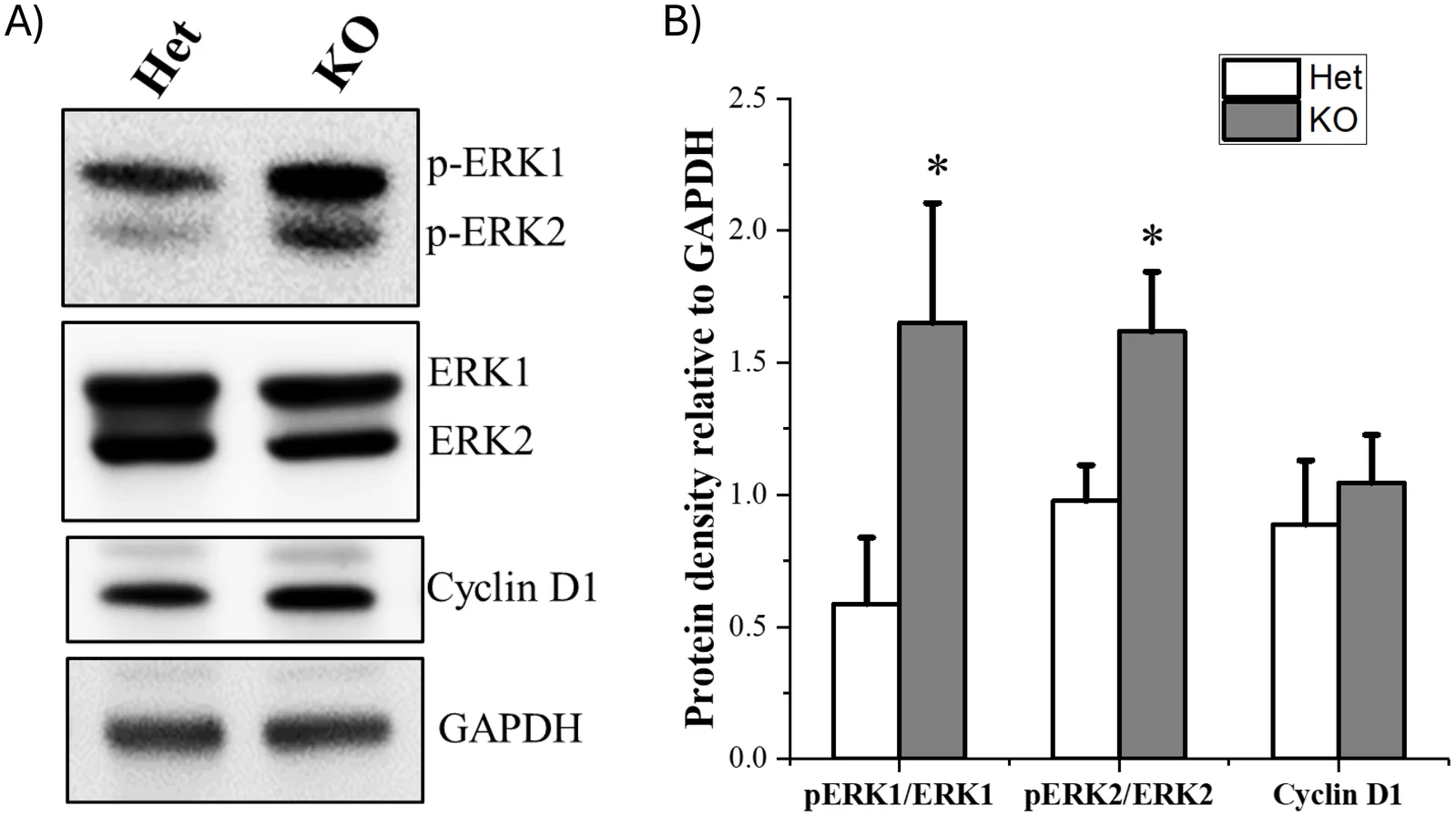
Lung lysate of N-WASP^LKO^/KRas^G12D^ mice had significantly higher levels of pERK1/2 compared to control mice lung lysate. **(A)** The lungs from N-WASP^LHet^/KRas^G12D^ (Het) and N-WASP^LKO^/KRas^G12D^ (KO) mice were isolated, minced and lysed in RIPA buffer. The protein extracts were cleared and 30ug was loaded on 10% SDS PAGE and probed with anti-pERK1/2, anti-ERK1/2 and anti-GAPDH. **(B)** The band intensity was quantified and normalized using GAPDH signal. Graph represents average value from 3 independently prepared lysates. (*p ≤ 0.05).

### 3.4 Transcriptomic profiling reveals coordinated transcriptional reprogramming upon N-WASP loss

To define the molecular programs underlying the enhanced tumor burden observed in N-WASP-deficient KRAS^G12D^ lungs, we performed bulk RNA sequencing on FACS-sorted type II alveolar epithelial cells (CD45^−^/EpCAM^+^) from N-WASP^LKO^/KRAS^G12D^ mice and genotype-matched controls. Unsupervised analyses revealed a profound transcriptional shift following N-WASP deletion. Principal component analysis showed clear segregation between knockout and control samples along the first principal component, indicating that loss of N-WASP induces a global and reproducible reprogramming of the lung tumor transcriptome (**Figure 4A**).

**Figure 4:**
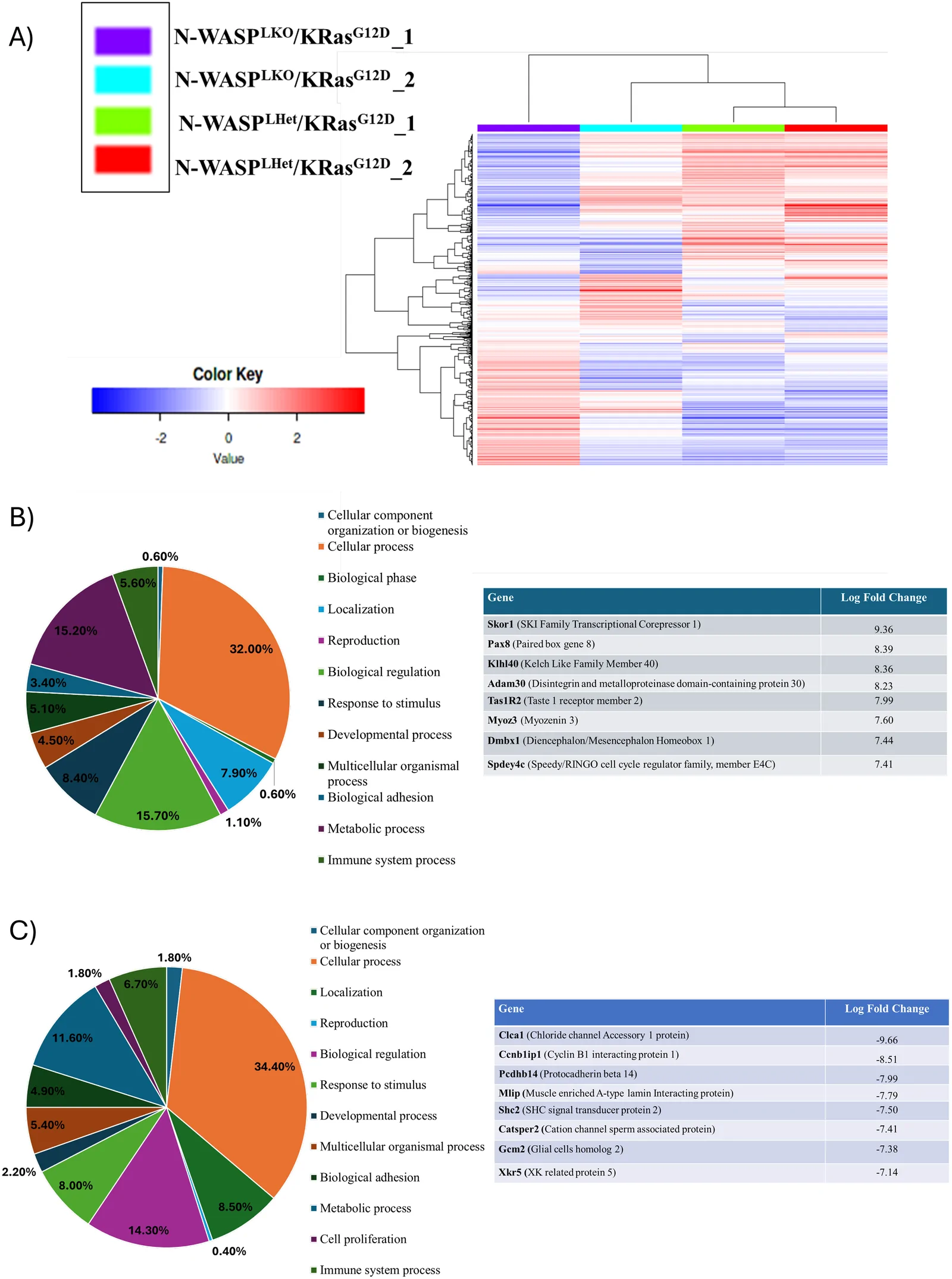
N-WASP knockout leads to upregulation of genes which promote cell proliferation. **(A)** Heatmap of different transcripts from Type II alveolar cells from N-WASP^LKO^/KRas^G12D^ and N-WASP^LHet^/KRas^G12D^ mice. The lungs from the mice were harvested, digested with dispase, collagenase and DNAse before sorting using anti-CD45 and PE anti-EpCam. Total RNA from the cells were isolated and the mRNA was enriched before subjecting to RNAseq analysis. The PCA clustering was generated based on normalized read counts of N-WASP^LKO^/KRas^G12D^ and N-WASP^LHet^/KRas^G12D^. The transcript levels are shown in different colors, transitioning from blue to red with increasing expressions. **(B)** Pie chart of functional classification of genes upregulated in N-WASP^LKO^/KRas^G12D^ compared to N-WASP^LHet^/KRas^G12D^ as determined by PANTHER analysis of RNA sequencing data and the top 8 genes which are upregulated. **(C)** Pie chart of functional classification of genes downregulated in N-WASP^LKO^/KRas^G12D^ compared to N-WASP^LHet^/KRas^G12D^ as determined by PANTHER analysis of RNA sequencing data and the top 8 genes which are downregulated.

Differential expression analysis identified 1,152 significantly upregulated (**Figure 4B**) and 721 downregulated genes (**Figure 4C**) in N-WASP-deficient lungs. PANTHER pathway analysis software was used to identify the pathways and list of top 8 genes that are highly upregulated in N-WASP^LKO^/KRas^G12D^ (**Figure 4B**) and top 8 genes that are highly downregulated in N-WASP^LKO^/KRas^G12D^ (**Figure 4C**). Hierarchical clustering of differentially expressed genes further demonstrated coherent gene expression patterns that clearly distinguished N-WASP^LKO^/KRAS^G12D^ samples from controls, consistent with a coordinated rather than stochastic transcriptional response. Functional enrichment analysis revealed that upregulated genes were predominantly associated with pathways linked to cell cycle progression, cytoskeletal remodeling, MAPK signaling, and oncogenic growth programs. These transcriptional changes align closely with the observed increase in ERK activation and tumor expansion, supporting a model in which N-WASP loss amplifies KRAS-driven mitogenic signaling outputs.

In contrast, downregulated genes were enriched for pathways involved in epithelial differentiation, cell–cell adhesion, and tissue homeostasis, including regulators of epithelial integrity and cytoskeletal anchoring. Suppression of these programs is consistent with the disrupted epithelial architecture and solid adenocarcinoma growth pattern observed histologically in N-WASP-deficient tumors. Together, these findings suggest that N-WASP loss shifts the balance from differentiated, structurally constrained epithelial states toward a more proliferative and plastic transcriptional landscape.

Collectively, transcriptomic data provides strong molecular support for the phenotypic consequences of N-WASP deletion and position N-WASP as a critical node linking actin cytoskeletal organization to KRAS-dependent transcriptional control. By coordinating signaling intensity and gene expression programs, N-WASP appears to restrain oncogenic reprogramming and tumor progression in lung adenocarcinoma.

### 3.5 Loss of N-WASP enhances lung cancer cell proliferation under 3D growth conditions

A549 cells are alveolar basal epithelial cell line derived from patient with lung adenocarcinoma with KRAS^G12S^ mutation (30). To define the functional role of N-WASP in lung cancer cell growth, stable N-WASP knockdown A549 cells (A549^KD^) and controls (A549^ctrl^) were generated using lentivirus generated using plasmid with N-WASP shRNA construct or control plasmid. Western blot analysis confirmed efficient depletion of N-WASP protein in A549^KD^ cells compared to control cell line A549^ctrl^ (**Figures 5A, B**). Given prior reports linking reduced N-WASP expression to poor prognosis in several cancers, including lung cancer, we first assessed whether N-WASP loss directly affects proliferative capacity. Because epithelial and tumor cells proliferate within a three-dimensional architecture *in vivo*, A549 cells were cultured as 3D spheroids, which promotes cell–cell cohesion and recreates spatial constraints found in tumors (31, 32).

**Figure 5:**
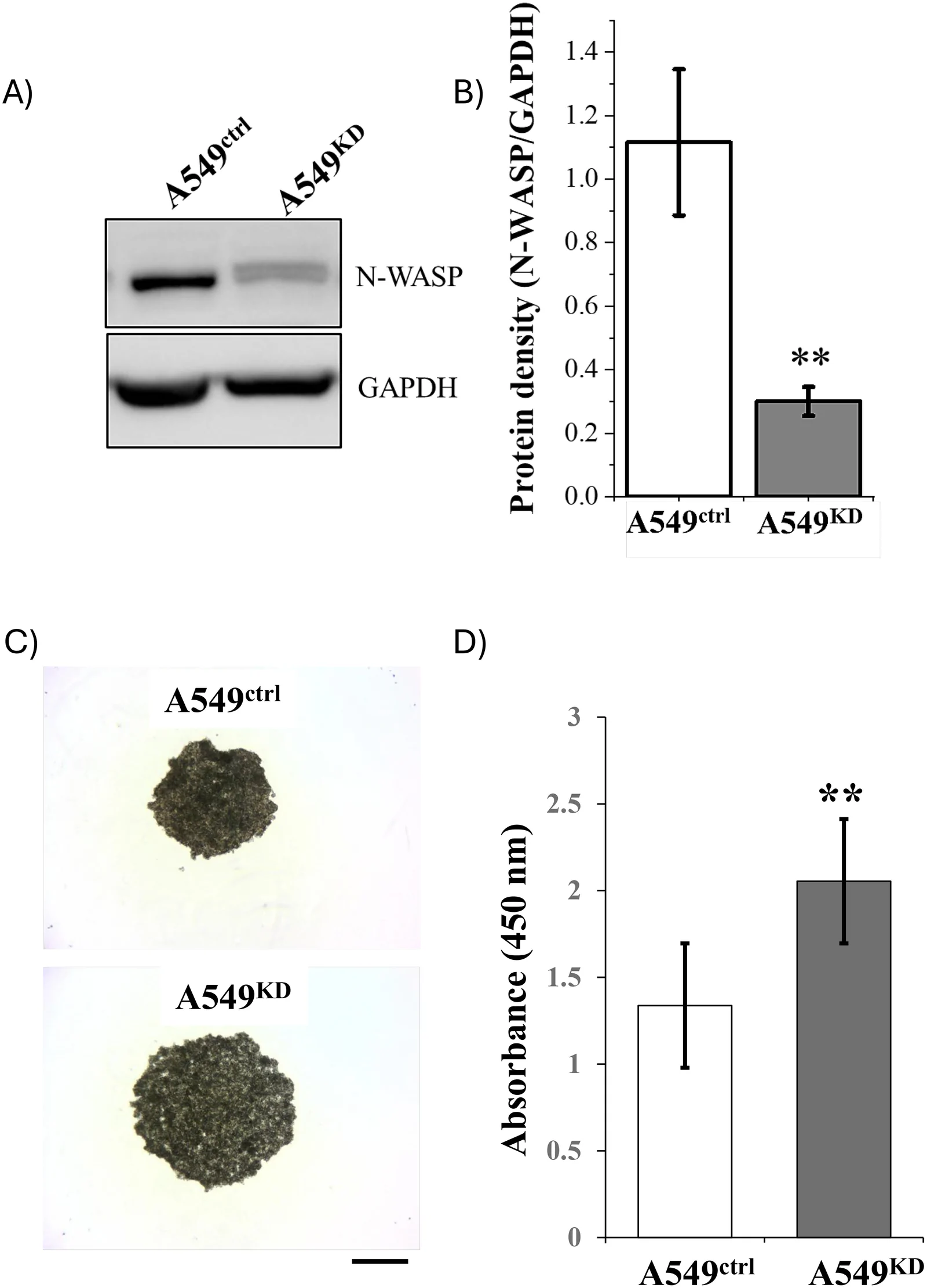
Knocking down of N-WASP expression in A549 cells enhances 3D cell proliferation. **(A)** A549 cells were infected either with control lentivirus or lentivirus expressing N-WASP specific shRNA (N-WASP shRNA). Cell lysates from A549^ctrl^ and A549^KD^ cells were prepared and equal amount of protein (30ug) was resolved on 10% SDS PAGE. The proteins were transferred to nitrocellulose membrane for western blot analysis using anti-N-WASP and anti-GAPDH antibodies. Experiments were performed in triplicates and densitometric quantification using ImageJ shows 80-85% reduction in N-WASP expression in A549^KD^ cells compared to A549^ctrl^. **(B)** Band intensity of N-WASP was quantified and normalized to GAPDH band intensity. There is a significant reduction of N-WASP in A549^KD^ cells compared to A549^ctrl^ cells. **(C)** Equal number (30X10^3^ cells/100 μl) of A549^ctrl^ and A549^KD^ were seeded in 96 well ULAP. The drops were then incubated for 72 hours, after which they were imaged. Scale Bar: 500 μm. **(D)** The A549^KD^ cells show significantly higher cell proliferation as determined using WST-8 assay. Graph represents data from three independent experiments, done in triplicates (Significance is indicated by stars, **p ≤ 0.01, ***p ≤ 0.001).

Under these conditions, A549^KD^ cells consistently formed large spheroids, whereas A549^ctrl^ cells formed smaller spheroids irregular clusters with visible gaps (**Figure 5C**). Notably, this spheroid-like growth and loss of epithelial organization closely mirrors the compact and poorly differentiated tumor architecture observed in N-WASP^LKO^/KRAS^G12D^ mice, suggesting a conserved role for N-WASP in maintaining epithelial structure across different experimental systems. Quantification of cell proliferation using MTT assay showed that A549^KD^ cells exhibited enhanced proliferation compared to the control cells (**Figure 5D**). To characterize the enhanced proliferation in 3D culture, we isolated protein lysate from A549^ctrl^ and A549^KD^ cells and carried out western blot analysis to detect levels of Erk1/2 and pErk1/2 (**Figure 6**). Quantification of the band intensities of Erk1/2 and pErk1/2 revealed that the A549^KD^ cells have increased levels of pErk2 similar to that observed in N-WASP^LKO^/KRAS^G12D^ mice. Together, these findings indicate that N-WASP restrains lung cancer cell proliferation in a context that requires cell–cell interactions, suggesting a role for cytoskeletal and adhesion-dependent growth control. These *in vitro* findings closely parallel the increased proliferation and disorganized growth observed upon N-WASP depletion *in vivo*, reinforcing the functional importance of N-WASP–mediated cytoskeletal control in restraining KRAS^G12D^-driven tumor progression.

**Figure 6:**
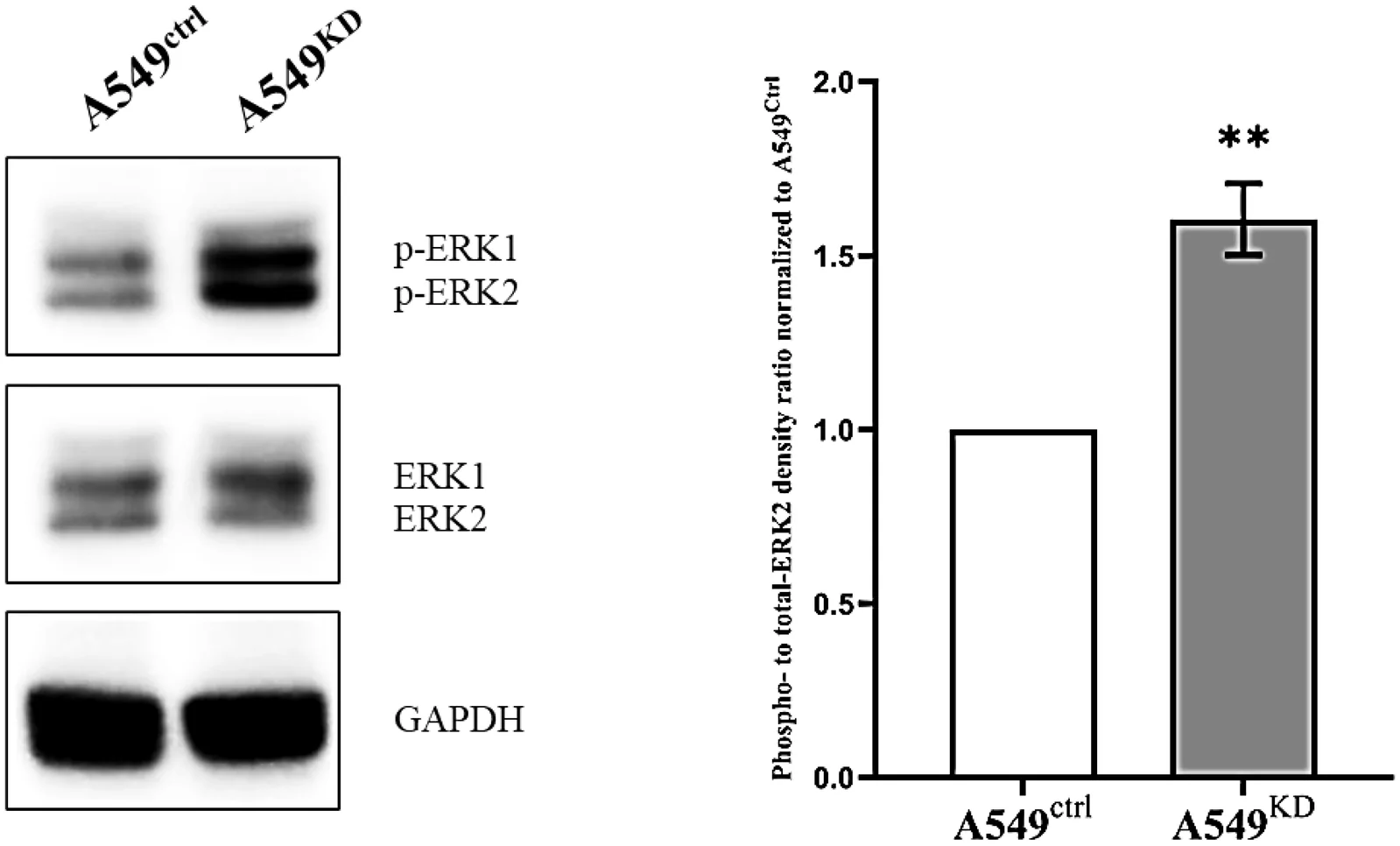
Knocking down of N-WASP expression in A549 cells enhances Erk2 phosphorylation. Cell lysates from A549^ctrl^ and A549^KD^ cells were prepared and equal amount of protein (30ug) was resolved on 10% SDS PAGE for western blot analysis using anti-Erk1/2, anti-pErk1/2 and anti-GAPDH antibodies. Experiments were performed in triplicates. Western blot quantification of pERK2 and total ERK2 levels. Knockdown of N-WASP in A549 cells significantly increased pERK2 levels compared to control (1.604-fold, p = 0.0020). ERK2 band intensities were first normalized to GAPDH and then to the corresponding A549^ctrl^ group within each biological replicate. Data are presented as fold change relative to A549^ctrl^. Each dot represents an independent biological replicate (n = 6). Statistical significance was determined using a two-tailed one-sample t-test comparing A549^KD^ against a theoretical mean of 1. Data are shown as mean ± SEM. *p < 0.05, **p < 0.01.

### 3.6 N-WASP knockdown disrupts E-cadherin–mediated adhesion in lung cancer spheroids

Given the spheroid morphology and increased proliferation of A549^KD^ cells, we hypothesized that N-WASP loss alters adherens junction integrity. E-cadherin is a central component of epithelial cell–cell adhesion and is known to suppress proliferation through contact inhibition mechanisms (33, 34). Importantly, previous studies have shown that N-WASP interacts with adherens junction components, including WIRE and E-cadherin, to stabilize epithelial junctions (35).

To test this, equal numbers of A549^ctrl^ and A549^KD^ cells were cultured as hanging drop spheroids for 48 hours and analyzed for E-cadherin expression. Western blot analysis revealed a significant reduction in total E-cadherin protein levels in A549^KD^ spheroids compared to controls (**Figure 7**). This loss of E-cadherin provides a plausible mechanistic explanation for the enhanced proliferation observed in N-WASP–deficient cells, as disruption of adhesion-mediated growth control can promote unchecked proliferation. These *in vitro* findings suggested that N-WASP loss may similarly enhance KRAS-driven tumorigenesis *in vivo* by weakening epithelial organization. We note that reduced E-cadherin alone is not sufficient evidence of a full epithelial-to-mesenchymal transition (EMT); classical EMT markers (N-cadherin, vimentin, Snail, Twist, Zeb1) were not examined here, and their inclusion in future work will help determine whether N-WASP loss drives a broader mesenchymal transition or a more limited disruption of adherens junction integrity.

**Figure 7:**
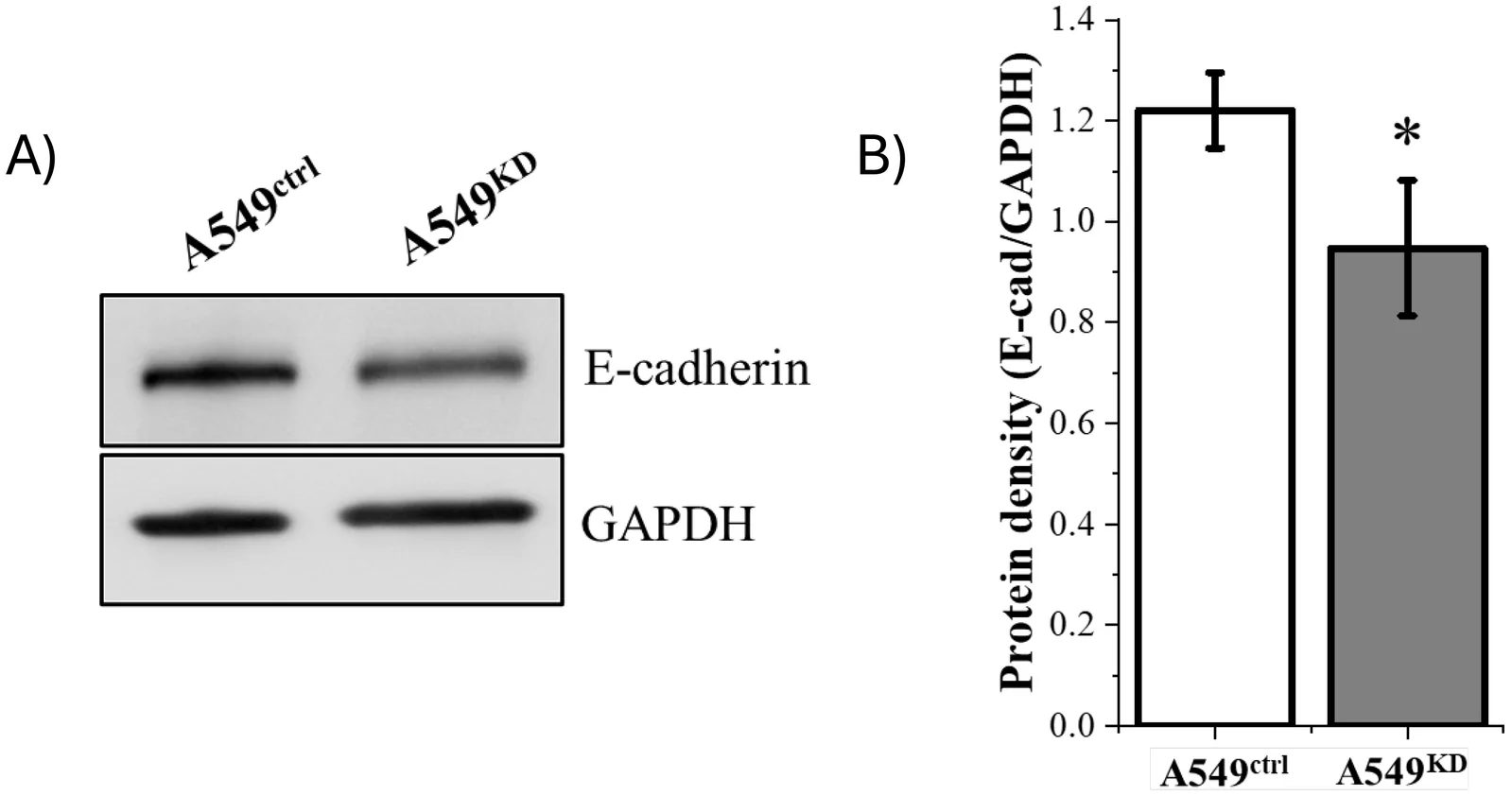
A549^KD^ cells have significantly reduced E-cadherin expression in 3D culture. Equal number of A549^ctrl^ and A549^KD^ cells were cultured in spheroid culture for 48 hours. The cells were lysed in RIPA buffer to extract the proteins. Equal amount of protein was loaded on 10% SDS PAGE. The expression was analyzed using anti-E-cad antibody. Image J was used to quantify the protein levels relative to GAPDH. Experiments were performed in triplicates (Significance is indicated by stars, *p ≤ 0.05).

### 3.7 Inhibition of Rho-associated protein kinase (ROCK) reduced cell proliferation of A549^KD^ cells

A549^KD^ cells exhibited increased cell proliferation in spheroid culture. Protein kinases function via phosphorylating other proteins in signal transduction pathways that regulate cell proliferation, differentiation and morphogenesis. This makes them as attractive therapeutic drug targets (36, 37). To identify the potential kinases that could be responsible for increased cell growth, a small-scale drug screening was carried out. A total of 192 kinase inhibitors (Synkinase Inhibitors and TargetMol Inhibitors) at their IC50 concentration were incubated with A549^KD^ cells in 96 well ULAP. Two kinase inhibitors GSK429286A and Staurosporine were found to decrease the cell proliferation by more than 30%. Staurosporine reduced cell proliferation by 49.9% but is known to be a very non-selective kinase inhibitor (38), thus we focused on GSK429286A which is a ROCK inhibitor as our previous studies have shown that WASP interacting Protein (WIP) regulates RhoA activity (39). We used a well-known ROCK inhibitor Y-27632 at 600nM on both A549^ctrl^ and A549^KD^ cells in spheroid culture. A549^ctrl^ and A549^KD^ cells were incubated with either DMSO or Y-27632 for 3 days, incubated with WST-8 for 2 hours and the absorbance at OD_450nm_ was measured. We found that Y-27632 caused a significant reduction in the proliferation of A549^KD^ cells and both A549^ctrl^ and A549^KD^ cells were found to proliferate at very similar rate when incubated with Y-27632 (**Figure 8**). This suggest that since ROCK inhibitor, specifically reduces the proliferation of A549^KD^ cells compared to A549^ctrl^ cells, so it was hypothesized that this kinase and its downstream effector proteins are more active or over-expressed in A549^KD^ cells which contributes to increased proliferation.

**Figure 8:**
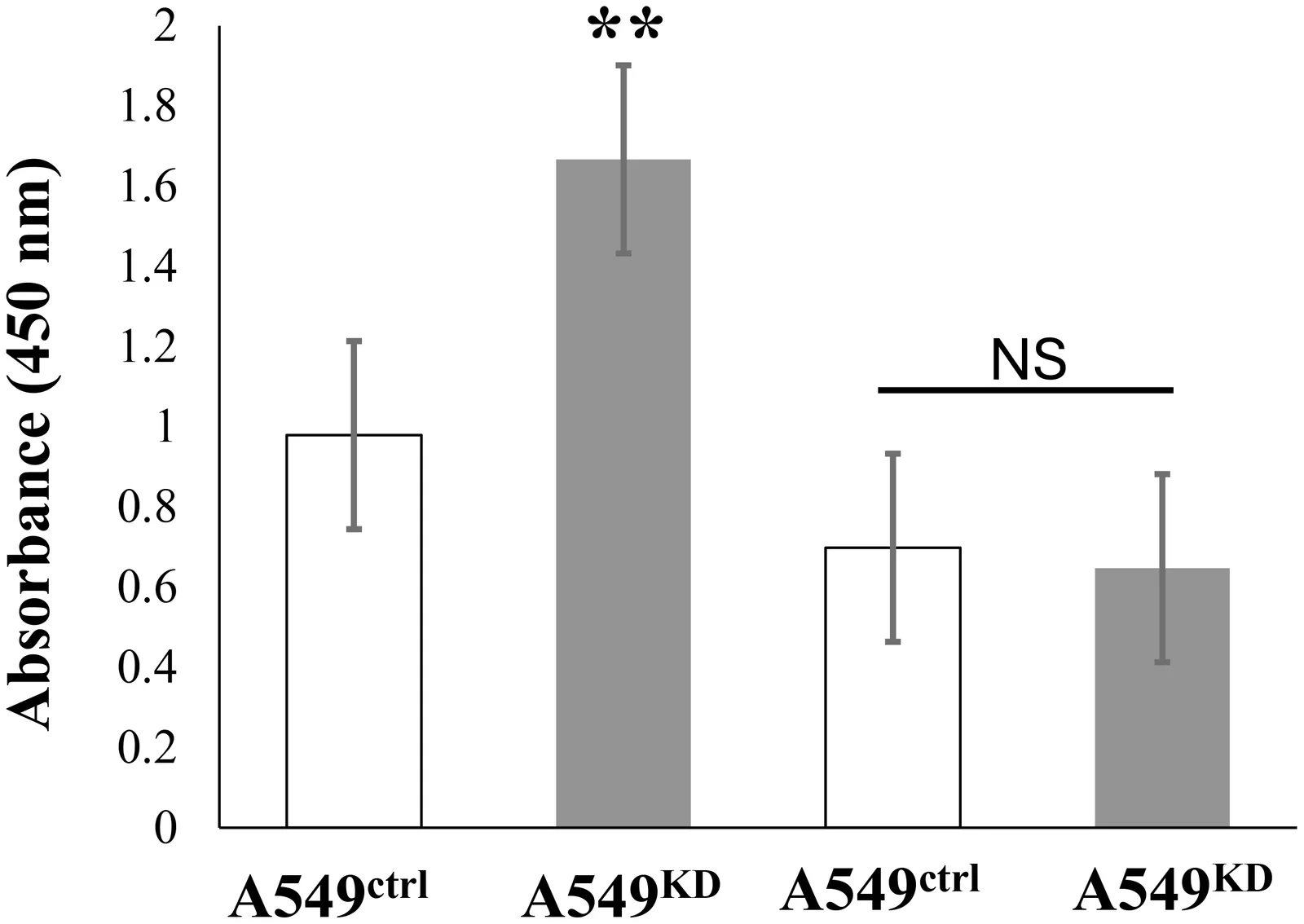
Inhibition of ROCK reduced proliferation of A549^KD^ cells to that of A549^ctrl^ cells. Equal number (30X10^3^ cells/100 μl) of A549^ctrl^ and A549^KD^ were seeded in 96 well ULAP and incubated with either DMSO or Y-27632 (600 nM) for 72 hours, after which they were incubated with 10 μl of WST-8 for 2 hours and the absorbance was measured. Graph represents data from three independent experiments, done in triplicates (Significance is indicated by stars, **p ≤ 0.01, ***p ≤ 0.001).

## 4 Discussion

N-WASP regulates the remodeling of actin cytoskeleton by regulating the activity of the Arp2/3 complex (40) and regulates cell-cell adhesion in a non-canonical pathway (35). Our study demonstrates that N-WASP acts as a critical suppressor of KRAS-driven lung adenocarcinoma, integrating cytoskeletal integrity, epithelial architecture, and oncogenic signaling. Conditional deletion of N-WASP in type II alveolar epithelial cells significantly increased tumor initiation, growth, and progression in KRAS^G12D^ mice. *In vitro* studies using N-WASP–deficient A549 cells mirrored these phenotypes, showing enhanced proliferation, disrupted actin cytoskeletal organization, and impaired cell–cell adhesion. Together, these results reveal that N-WASP restrains KRAS-driven tumorigenesis by coordinating both structural and signaling networks in epithelial cells.

Mechanistically, loss of N-WASP enhanced ERK1/2 phosphorylation *in vivo* lung tumors, providing a molecular explanation for the hyperproliferative phenotype seen in mice. Interestingly, cyclin D1 levels did not proportionally increase, suggesting that N-WASP regulates the spatial and compartmentalized fidelity of MAPK signaling, rather than simply amplifying pathway activity. This is consistent with prior report that cytoskeletal scaffolds orchestrate precise subcellular signaling (29), positioning N-WASP as a structural checkpoint that maintains proper signal transmission during tumorigenesis. Kinase inhibitor screening identified two kinase inhibitors GSK429286A (ROCK inhibitor) and Staurosporine which decreased the cell proliferation by more than 30%. Staurosporine is not a very non-selective kinase inhibitor thus we focused on inhibition of ROCK using Y-27632 which reduced the cell proliferation of A549^KD^ cells to that of A549^ctrl^ cells suggesting that the increased cell proliferation of A549^KD^ cells could be due to increased activity of Rho/ROCK in A549^KD^ cells. We note that the ERK1/2 activation data remain correlative at this stage, establishing direct causality will require MEK-inhibitor treatment, N-WASP re-expression (rescue) experiments, and mapping of N-WASP interactions with KRAS-ERK pathway effectors. Notably, Rho/ROCK signaling has been shown to sustain ERK1/2 activation and drive proliferation in other cancer models, and combined ROCK and MEK inhibition produces a more pronounced suppression of ERK1/2 phosphorylation than MEK inhibition alone (41). This raises the possibility that the elevated Rho/ROCK activity identified in our kinase inhibitor screen contributes to, or reinforces, the increased pERK1/2 observed in N-WASP-deficient tumors and A549-KD cells, suggesting a Rho/ROCK-ERK signaling axis downstream of N-WASP loss; direct testing of this link, for example by measuring pERK1/2 following ROCK inhibition, is an important next step.

The tumor-suppressive role of N-WASP is context-dependent, as highlighted by studies in other epithelial tissues. In the skin, ablation of N-WASP using Cytokeratin 14 induced atopic-dermatitis-like inflammation like skin with epidermal thickening, barrier defects and immune cell infiltration (20). The epidermal thickening was due to keratinocyte hyperproliferation. In KRAS-driven SCC mouse models, activation of KRAS^G12D^ in keratinocytes triggers SCC formation, redundant skin, hair loss, and papillomas (42). These observations suggest that N-WASP constrains KRAS oncogenic potential across epithelial tissues by preserving actin architecture, cell–cell adhesion, and epithelial homeostasis. The parallels between lung adenocarcinoma and skin SCC models emphasize that N-WASP’s tumor-suppressive function relies on maintaining structural integrity within epithelial compartments.

Transcriptomic profiling of N-WASP–deficient lungs revealed a coordinated transcriptional reprogramming: upregulation of genes linked to proliferation, cytoskeletal remodeling, and MAPK signaling, and downregulation of epithelial differentiation and adhesion programs. This shift corresponds with the solid, poorly differentiated tumor histology observed in N-WASP^LKO^/KRAS^G12D^ lungs and reflects a broader transition toward a proliferative, plastic epithelial state. Loss of E-cadherin in spheroid cultures further illustrates how disruption of adhesion-mediated growth control can amplify KRAS-driven proliferation, reinforcing the idea that cytoskeletal organization is a central barrier to tumor expansion.

Skor1 is known to negatively regulate anti-proliferative TGF-β pathway (43), Pax8 has been identified as an oncogenic transcription factor in renal cell carcinoma (44), Klhl40 possess a N-terminal domain which regulate cell cycle (45), Adam30 regulate cell adhesion and migration (46), Tas1R2 increase glucose absorption (47), Dmbx1 has found to be over-expressed in lung adenocarcinoma patients (48). Clca1 found to be significantly reduced compared to normal tissue (49, 50), Ccnb1ip1 expression has been found to be reduced in breast and lung cancer (51), Expression of Pcdhb14 was found reduced in CRC patients (52, 53).

Clinical correlations support the context-dependent role of N-WASP. High N-WASP expression is associated with poor prognosis in pancreatic, liver, and cervical cancers (54–56), whereas low N-WASP levels correlate with poor outcomes in early-stage lung, colorectal, and renal cancers (14, 16, 57). Our data support the latter scenario: during early KRAS-driven lung tumorigenesis, N-WASP functions as a barrier to malignant expansion. These findings suggest that N-WASP’s role as a tumor suppressor is most pronounced during the initiation and early progression stages, with its loss unmasking the full proliferative potential of oncogenic KRAS.

Beyond mechanistic insights, our study highlights therapeutic implications. Targeting pathways that restore N-WASP function or mimic its cytoskeletal and adhesion-regulatory activity could constrain KRAS-driven tumor growth. Such strategies may complement emerging therapies targeting KRAS or downstream MAPK signaling, offering a multi-layered approach to a historically refractory lung cancer subtype. Furthermore, the parallels observed between lung and skin epithelia indicate that N-WASP-dependent mechanisms may be broadly relevant across epithelial cancers harboring oncogenic KRAS mutations. We emphasize that this therapeutic framing is hypothesis-generating rather than an immediate proposal for clinical translation, given that N-WASP shows opposing prognostic associations across tumor types (14, 16, 49–52) and any such strategy would require tissue- and stage-specific validation.

Several limitations should be noted. Mice were analyzed at a single, fixed timepoint; longitudinal survival analysis and assessment of invasion/metastasis will be needed to confirm long-term relevance. Because pERK1/2 and cyclin D1 were measured in whole-lung lysates, tumor-region microdissection or immunohistochemical quantification, together with blinded histological grading and Ki67 indices, would provide more precise evidence of malignancy. N-WASP re-expression (rescue) experiments in A549^KD^ cells will be needed to establish causality for the phenotypes described here. We also did not examine the tumor microenvironment, additional KRAS-mutant allelic variants (e.g., G12C, G12V, G13D), or potential compensation by other WASP-family proteins (WASP, WAVE1-3, WHAMM/JMY); addressing these will help clarify the generalizability and completeness of the phenotype described and are priorities for future work.

## 5 Conclusion

In summary, N-WASP restrains KRAS-driven lung adenocarcinoma by integrating cytoskeletal dynamics, epithelial architecture, and MAPK signaling. Its loss accelerates tumor initiation and progression, promotes a proliferative transcriptional program, and disrupts epithelial homeostasis. These findings position N-WASP as a critical structural and signaling checkpoint in KRAS-mutant cancers and underscore cytoskeletal regulation as a promising, yet underexplored, target for therapeutic intervention. Further studies dissecting the interplay between N-WASP, adhesion complexes, and spatial MAPK signaling will be crucial to harness this pathway clinically.

## Data Availability

The data presented in the study are deposited in the Gene Expression Omnibus (GEO) repository, accession number GSE343586.

## Supplementary material

Supplementary Figure 1 Schematic diagram of N-WASP alleles and genotyping using PCR. N-WASP specific PCR primers produce a PCR product of 500bp for WT N-WASP gene and 700bp PCR product for floxed N-WASP, hence (N-WASP^WT/WT)^, produce a single 500bp band while heterozygous mice (N-WASP^fl/WT^), produce 2 bands of 700bp and 500bp while homozygous mice (N-WASP^fl/fl^), produce a single band of 700bp. The presence Cre is checked using Sftpc Cre primers (sF1, sF2 & sR1) and produced a PCR product of 320bp when there is no Cre and 200bp PCR product when there is a Cre.

Supplementary Figure 2 Lung tumors were characterized as adenocarcinoma by staining with proSP-C. The lungs from N-WASP^LKO^/KRas^G12D^ (KO) and N-WASP^LHet^/KRas^G12D^ (Het ctrl) were isolated, fixed, dehydrated and embedded in paraffin. 5µm thickness sections were cut and proSP-C immunostaining was performed. For antigen retrieval, slides were boiled in citrate buffer and overnight proSP-C primary Ab incubation was carried out. The following day, secondary Ab incubation and DAPI staining were performed. The slides were mounted, and images were captured at 40X oil lens using Olympus IX 51 microscope. The staining was performed on n=3 per group. The sorted epithelial cells were stained with ProSP-C, marker of Type II alveolar cells.
