# Supplementary material for: N-WASP deficiency enhances oncogenic KRAS-mediated lung adenocarcinoma progression in mice
Source: Front Oncol. 2026 Oct 8;16:1868586. doi: 10.3389/fonc.2026.1868586 (PMC13686841; doi:10.3389/fonc.2026.1868586)
Supplement: Supplementary file 1 [file DataSheet1.pdf]

N-WASP deficiency enhances oncogenic KRAS-mediated lung adenocarcinoma progression in mice

Apoorva Verma[1], Nanyang Li[1], Pazhanichamy Kalailingam[1], Chaiyoung Kim[1], Ankur Sharma[1], Ying Hui Lee[1] and Thirumaran Thanabalu[1]*

[1]School of Biological Sciences, Nanyang Technological University, 60 Nanyang Drive, Singapore 637551, Singapore

Present address for Apoorva Verma: AstraZeneca Singapore Pte Ltd, 10 Kallang Avenue, Singapore 339510, Singapore

Present address for Pazhanichamy Kalailingam: Massachusetts General Hospital, Harvard Medical School, Boston, MA 02114, USA

*Correspondence: thirumaran@ntu.edu.sg; Tel.: +65-6316-2832

Keywords: KRAS Lung Cancer, Transgenic mice, N-WASP, Erk2 signaling, Actin Cytoskeleton, Tumor Suppressor

Supplementary Figure

Figure S1: Schematic diagram of N-WASP alleles and genotyping using PCR.

N-WASP specific PCR primers produce a PCR product of 500bp for WT N-WASP gene and 700bp PCR product for floxed N-WASP, hence (N-WASP$^{WT/WT)}$, produce a single 500bp band while heterozygous mice (N-WASP$^{fl/WT}$), produce 2 bands of 700bp and 500bp while homozygous mice (N-WASP$^{fl/fl}$), produce a single band of 700bp. The presence Cre is checked using Sftpc Cre primers (sF1, sF2 & sR1) and produced a PCR product of 320bp when there is no Cre and 200bp PCR product when there is a Cre.

Figure S2: Lung tumors were characterized as adenocarcinoma by staining with proSP-C. The lungs from N-WASP^{LKO}/KRas^{G12D} (KO) and N-WASP^{LHet}/KRas^{G12D} (Het ctrl) were isolated, fixed, dehydrated and embedded in paraffin. 5µm thickness sections were cut and proSP-C immunostaining was performed. For antigen retrieval, slides were boiled in citrate buffer and overnight proSP-C primary Ab incubation was carried out. The following day, secondary Ab incubation and DAPI staining were performed. The slides were mounted, and images were captured at 40X oil lens using Olympus IX 51 microscope. The staining was performed on n=3 per group. The sorted epithelial cells were stained with ProSP-C, marker of Type II alveolar cells.

Fig. S1

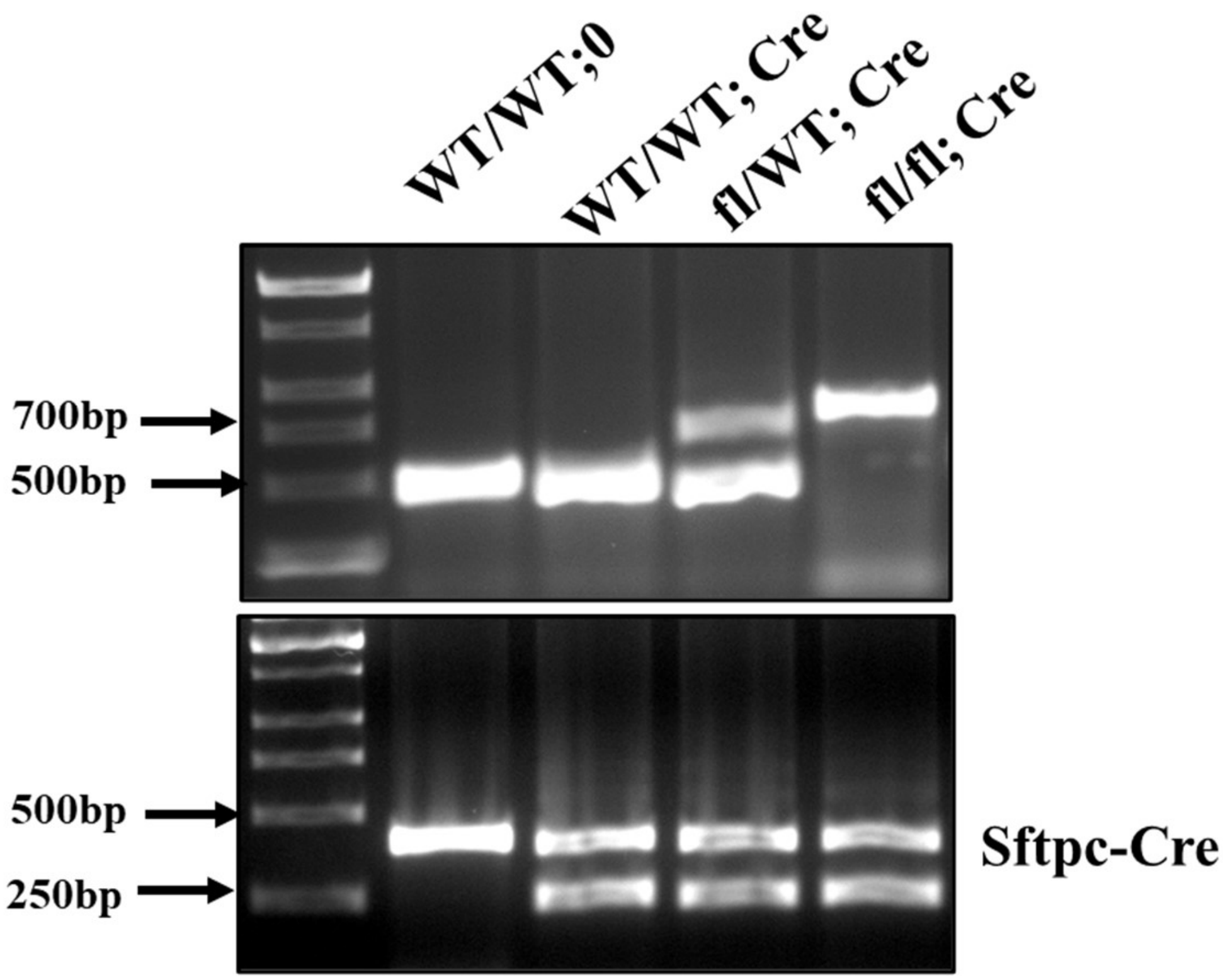

WT/WT;0
WT/WT; Cre
fl/WT; Cre
fl/fl; Cre
700bp
500bp
500bp
250bp
Sftpc-Cre

Fig. S2

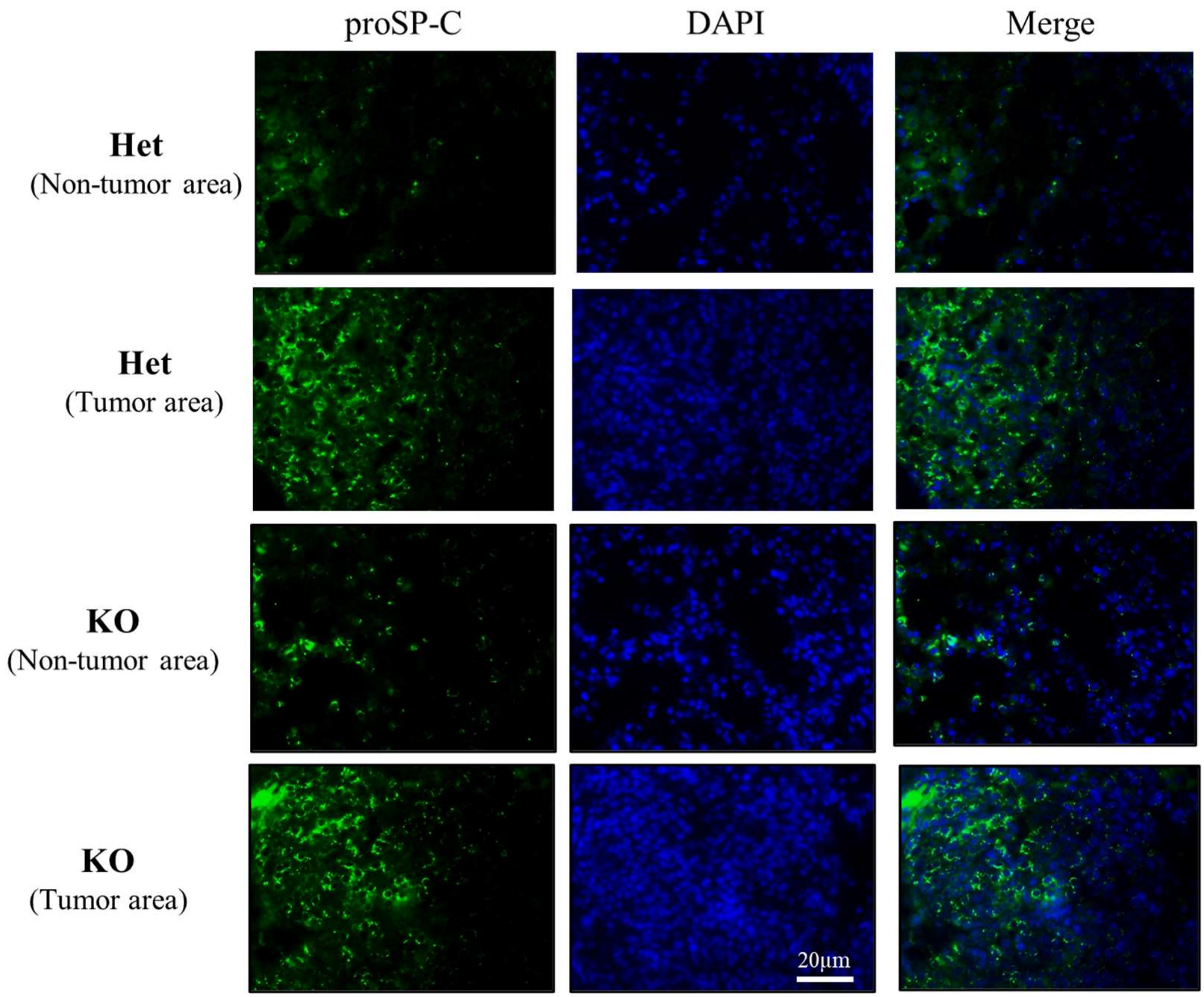

proSP-C
DAPI
Merge
Het
(Non-tumor area)
Het
(Tumor area)
KO
(Non-tumor area)
KO
(Tumor area)
20μm
